# Reasoning by exclusion in the kea (*Nestor notabilis*)

**DOI:** 10.1007/s10071-016-0998-x

**Published:** 2016-05-21

**Authors:** Mark O’Hara, Raoul Schwing, Ira Federspiel, Gyula K. Gajdon, Ludwig Huber

**Affiliations:** 1 Department of Cognitive Biology, University of Vienna, Vienna, Austria; 2 Messerli Research Institute, University of Veterinary Medicine Vienna, Medical University Vienna, University of Vienna, Vienna, Austria

**Keywords:** Avian cognition, Categorisation, Inference by exclusion, Touch screen, Kea (*Nestor notabilis*)

## Abstract

Reasoning by exclusion, i.e. the ability to understand that if there are only two possibilities and if it is not A, it must be B, has been a topic of great interest in recent comparative cognition research. Many studies have investigated this ability, employing different methods, but rarely exploring concurrent decision processes underlying choice behaviour of non-human animals encountering inconsistent or incomplete information. Here, we employed a novel training and test method in order to perform an in-depth analysis of the underlying processes. Importantly, to discourage the explorative behaviour of the kea, a highly neophilic species, the training included a large amount of novel, unrewarded stimuli. The subsequent test consisted of 30 sessions with different sequences of four test trials. In these test trials, we confronted the kea with novel stimuli that were paired with either the rewarded or unrewarded training stimuli or with the novel stimuli of previous test trials. Once habituated to novelty, eight out of fourteen kea tested responded to novel stimuli by inferring their contingency via logical exclusion of the alternative. One individual inferred predominantly in this way, while other response strategies, such as one trial learning, stimulus preferences and avoiding the negative stimulus also guided the responses of the remaining individuals. Interestingly, the difficulty of the task had no influence on the test performance. We discuss the implications of these findings for the current hypotheses about the emergence of inferential reasoning in some avian species, considering causal links to brain size, feeding ecology and social complexity.

## Introduction

The field of cognition research attempts to unravel the mechanisms underlying the adaptive modification of behaviour through individual or social learning. A topic of great interest within comparative cognition is whether non-human animals are able to solve novel problems in a quick and beneficial way despite having incomplete information. One form of dealing with uncertainty when facing novel situations is to reason about known instances and logically exclude alternatives (Call 2006). The common approach to investigating such cognitive abilities is to devise tasks that, by systematically excluding alternative explanations, allow us to conclude if a certain ability is present or not (e.g. Call 2006; Aust et al. 2008; Schloegl et al. 2009a, b; Mikolasch et al. 2012; Shaw et al. 2013; Jelbert et al. 2015). However, behaviours other than the one of interest are rarely considered and often dismissed as not noteworthy, thus creating a very binary ‘all or nothing’ approach to the mental capacities of animals.

An earlier experimental design by Aust et al. (2008) already successfully investigated exclusion skills in pigeons (*Columba livia*), dogs (*Canis lupus familiaris*) and humans using a touch screen apparatus. The benefits of using such a touch screen to present abstract tasks include efficient data collection, reduction of biases through cues given by the experimenter and applicability to a large variety of different species (e.g. Steurer et al. 2012). However, while the experimental design of Aust et al. (2008) controlled for responses based on neophilia, it was not intended to test for inference by exclusion in neophilic species. A recent study of O’Hara et al. (2015a) adapted the design of Aust et al. (2008) to overcome previous limitations with respect to neophilia and shift the focus from an ‘all or nothing’ approach to a more holistic analysis of choice behaviour. This was achieved by implementing a test procedure, which considered all possible response patterns and attributed these to the simplest cognitive processes underlying choice behaviour (henceforth referred to as response strategies). Additional habituation to novelty during the training proved effective at controlling for neophilic responses of very investigative species such as the Goffin cockatoo (*Cacatua goffiniana*). Not only could the authors show, with this set-up, that Goffins were able to base choices on inference by exclusion, but also highlight the importance of other response strategies.

The kea (*Nestor notabilis*) is a parrot species endemic to the alpine and subalpine regions of the South Island of New Zealand. Sub-adult individuals form flocks of up to 20 individuals, whereas adults pair for life, forming family groups and joining larger groups occasionally at foraging sites (Clarke 1970; Diamond and Bond 1999). Thus, the kea’s social structure may be best described as fission–fusion-like. Their climatically harsh environment and low predatory risk are believed to have caused the kea’s explorative and curious nature (Diamond and Bond 1999; Huber and Gajdon 2006; Auersperg et al. 2011; O’Hara et al. 2015b). It is this neophilia we suspect to have overshadowed exclusion skills in a previous attempt to compare inferential abilities in ravens (*Corvus corax*) and kea (Schloegl et al. 2009b). Schloegl and colleagues used a foraging task, originally devised by Call and Carpenter (2001), in which either straight or bent tubes were baited with food items and presented simultaneously to the individuals. While ravens discarded empty, straight tubes that were oriented towards them and thus provided visual information of the presence or absence of food, kea investigated empty tubes from both sides in approximately a third of all trials before choosing the ‘correct’ tube. These results led Schloegl et al. (2009a, b) to conclude that competitiveness and food-storing behaviour may have promoted the raven’s exclusion skills, while the feeding ecology of the kea, an extractive forager (Brejaart 1988; Diamond and Bond 1999), led to extensive search behaviour, which at a first glance seems inefficient sometimes (O’Hara et al. 2012, 2015b; Gajdon et al. 2013; Greer et al. 2015). However, extractive foraging has been suggested to also promote exclusion skills in primates (Bräuer et al. 2006; Paukner et al. 2009; Marsh et al. 2015), as it may require individuals to infer the location of hidden food, whereas others (e.g. Petit et al. 2015) have argued that social complexity was the driving force for the ability to infer by exclusion.

Neophilia, as the predisposition to explore novel objects (Greenberg 2003), has been identified as an important factor accounting for biased test results especially in kea (O’Hara et al. 2012). Greenberg and Mettke-Hofmann (2001) have established a two-factor model that allows for predictions to be made based upon which ecological circumstances neophilia and exploration may be expected for different species. They conclude that a complex environment and low predation risk promote neophilic tendencies. Species inhabiting islands, such as the kea and Goffin cockatoos, seem more prone to investigate novel items, as on islands exploration may be very rewarding and at the same time not very costly (Greenberg and Mettke-Hofmann 2001). One way to overcome such tendencies in behavioural testing is to habituate individuals to novelty. Once individuals have sufficiently explored and habituated to a novel set-up they may exhibit cognitively more demanding abilities (Gajdon et al. 2013).

Kea have been shown to be exceptional problem solvers in the technical domain (Werdenich and Huber 2006; Huber and Gajdon 2006; Auersperg et al. 2009, 2011) and possess a large brain size compared to their body weight (brain weight/body weight = 0.015; see Iwaniuk et al. 2005), comparable to that of ravens (brain weight/body weight = 0.011; see Sol et al. 2010). Thus, corvids and parrots constitute prime candidates for advanced cognitive abilities among birds (Lefebvre et al. 2004; Iwaniuk et al. 2005; Roth and Dicke 2005). Therefore, we hypothesise that neophilia overruled the capacity to reason by exclusion rather than preclude such abilities in a previous study (Schloegl et al. 2009b).

Exclusion skills in non-human animals may have different origins. Several corvid researchers have proposed the emergence of exclusion skills in these birds as an adaption to their specialised feeding ecology (Schloegl et al. 2009b; Mikolasch et al. 2012), in support of the adaptive specialisation hypothesis (Krebs 1990; de Kort and Clayton 2006). Some primatologists, however, argued that in primates these skills have emerged as an adaptation to socially complex situations (Petit et al. 2015). Further, Pepperberg et al. (2013) have suggested that these skills are a fundamental cognitive ability and a marker of general intelligence.

If we accept relative brain size (defined as size of cortical or equivalent structures in relation to the overall brain size) as a reasonable indicator of cognitive abilities, and one marker of such to be the ability to reason based on exclusion, as suggested by Pepperberg et al. (2013), we would predict the kea to be capable of solving problems of this nature, if confounding behavioural predispositions (e.g. neophilia) are controlled for. The alternative hypothesis that exclusion abilities constitute an adaption to a specialised feeding ecology of food-storing birds (Schloegl et al. 2009a, b; Schloegl 2011; Mikolasch et al. 2012) would predict this ability to be absent in the kea. Unless it has evolved independently (at least twice) in birds, under different evolutionary pressures, kea are not storing food and are only distantly related to corvids.

Studies on exclusion performance in animals have also discussed the effect of task difficulty on the ability to make inferences (Grether and Maslow 1937; Marsh et al. 2015). Therefore, in addition to the possible effects of neophilia we investigated the effect of cognitive load (Sweller 1988) on task performance. By modifying the original procedure (O’Hara et al. 2015a), we introduced a group that received the test trials in an alternative sequence, which should result in a decrease in test trial spacing, thus benefiting the working memory and could be considered computationally less demanding (Barrouillet et al. 2007).

## Materials and methods

### Ethical statement

The Animal Ethics and Experimentation Board of the Faculty of Life Sciences at the University of Vienna was informed and approved this study (Reference number: 2015-006).

### Test subjects

Fourteen kea of different ages (one juvenile, 1 year of age; one sub-adult, 4 years of age; and twelve adults, mean age 9 ± 3 years) and sexes (nine males, five females) housed in a large group aviary (approx. 520 m^2^) at the Haidlhof Research Station near Vienna, Austria, participated in this study (see Table 1). The group was fed three times per day with vegetables, fruits, protein and seeds, and water was available ad libitum. All individuals were familiar with the touch screen and had participated in prior touch screen tasks (O’Hara et al. 2015a).

**Table 1:** Overview of individuals participating in this study

Individual	Sex	Age group	Rearing	Experience	Group
Anu	♂	Adult	Hand	No	B
Coco	♀	Adult	Hand	No	B
Elvira	♀	Adult	Parent	No	A
Frowin	♂	Adult	Parent	Yes	B
John	♂	Adult	Parent	Yes	A
Kermit	♂	Adult	Hand	Yes	A
Lilly	♀	Adult	Hand	No	A
Linus	♂	Adult	Hand	Yes	B
Papu	♀	Juvenile	Hand	No	A
Paul	♂	Sub-adult	Parent	No	B
Pick	♂	Adult	Hand	Yes	B
Roku	♂	Adult	Parent	No	A
Sunny	♀	Adult	Hand	No	A
Willy	♀	Adult	Hand	No	B

Names of individuals participating in this experiment along with sex (♂ for males, ♀ for females) and respective age group; rearing indicates if individuals were hand raised or parent raised; experience denotes whether or not individuals had participated in the previous exclusion study by Schloegl et al. (2009a, b); group refers to experimental group which each individual was assigned to

### Apparatus

We used an adapted version of the operant conditioning system described in detail by Steurer et al. (2012). The apparatus consisted of a touch-sensitive screen (304 mm × 228 mm display area, 381 mm diagonal, 1024 × 768 pixels), CPU and automatic rewarding system; rewards were delivered centrally, 90 mm below the lower edge of the screen. Individuals had easy access to the screen and reward tray by standing on a platform (400 × 700 mm), which was installed directly below the reward tray. To avoid reflections of sunlight, wooden panels were attached above and behind this platform, nonetheless allowing the birds to retreat to the side at any time. The program used for testing was ‘CognitionLab’ (version 1.9; see Steurer et al. 2012, for a detailed description). An arbitrary collection of licence and restriction-free clip arts, downloaded from the Open Clip Art Library (http://www.openclipart.org/), was standardised to generate images on white background, measuring 70 by 70 pixels, adapted for equal overall brightness, and converted to Portable Network Graphic (png) files using Fiji (ImageJ 1.49e, http://imagej.nih.gov/ij; ImageJ 2.0.0-rc-9, http://developer.imagej.net/). Two stimuli were presented simultaneously within each trial, on the central horizontal axis of the screen, 341 and 682 pixels, respectively, away from the left side of the screen.

### Procedure

The procedure followed the protocol of O’Hara et al. (2015a), with two exceptions: thirty test sessions were provided instead of 25, and testing was split into two groups, with one group exactly following the procedure of O’Hara et al. (2015a) and the second group only differing in the sequence of test trials. We give a short summary of the general procedure below and elaborate more on the differences between the groups.

#### Training

Each trial involved the simultaneous presentation of two stimuli, of which one was rewarded (S+) and one was unrewarded (S−). While touching the S+ led to the delivery of an eighth of a peanut seed, touching the S− led to a 2-s timeout during which the screen went blank. Each session consisted of 20 trials, 18 training (baseline) trials and two randomly intermixed ‘novelty’ trials. For each subject, we selected a different pair of images (S+ and S−) that would remain the same throughout all training trials. In the novelty trials, the negative training stimulus was replaced by a novel stimulus, being a different one in each novelty trial. The stimuli were chosen and assigned randomly to each individual from a pool of 190 possible icons. The novelty trials were intended to habituate the kea to novel stimuli and to reduce the neophilic tendencies to explore a novel stimulus. However, this procedure was not aimed at training inhibition towards novelty in general; therefore, training did not continue until the subjects would significantly choose the familiar stimulus over novel ones. For this reason, we continued training until individuals ceased to respond to both novel stimuli instead of choosing the S+ for two consecutive sessions (Criterion 1). To ensure that the birds had learned the basic discrimination, they were required to choose correctly in more than 80 % (15/18) of the training trials for two consecutive sessions (Criterion 2).

#### Testing

Once both criteria were met, 30 sessions of testing followed. Each session included four test trials intermixed within 16 training trials. In order to prevent a violation of expectancy in test trials, stimuli with positive contingency were rewarded (S+), whereas responses to stimuli with negative contingency (S−) were immediately aborted (no sound) and followed by the next training trial. Because this differential rewarding of test stimuli could lead to absolute stimulus learning, we used novel test stimuli for each test session.

**Fig. 1:**
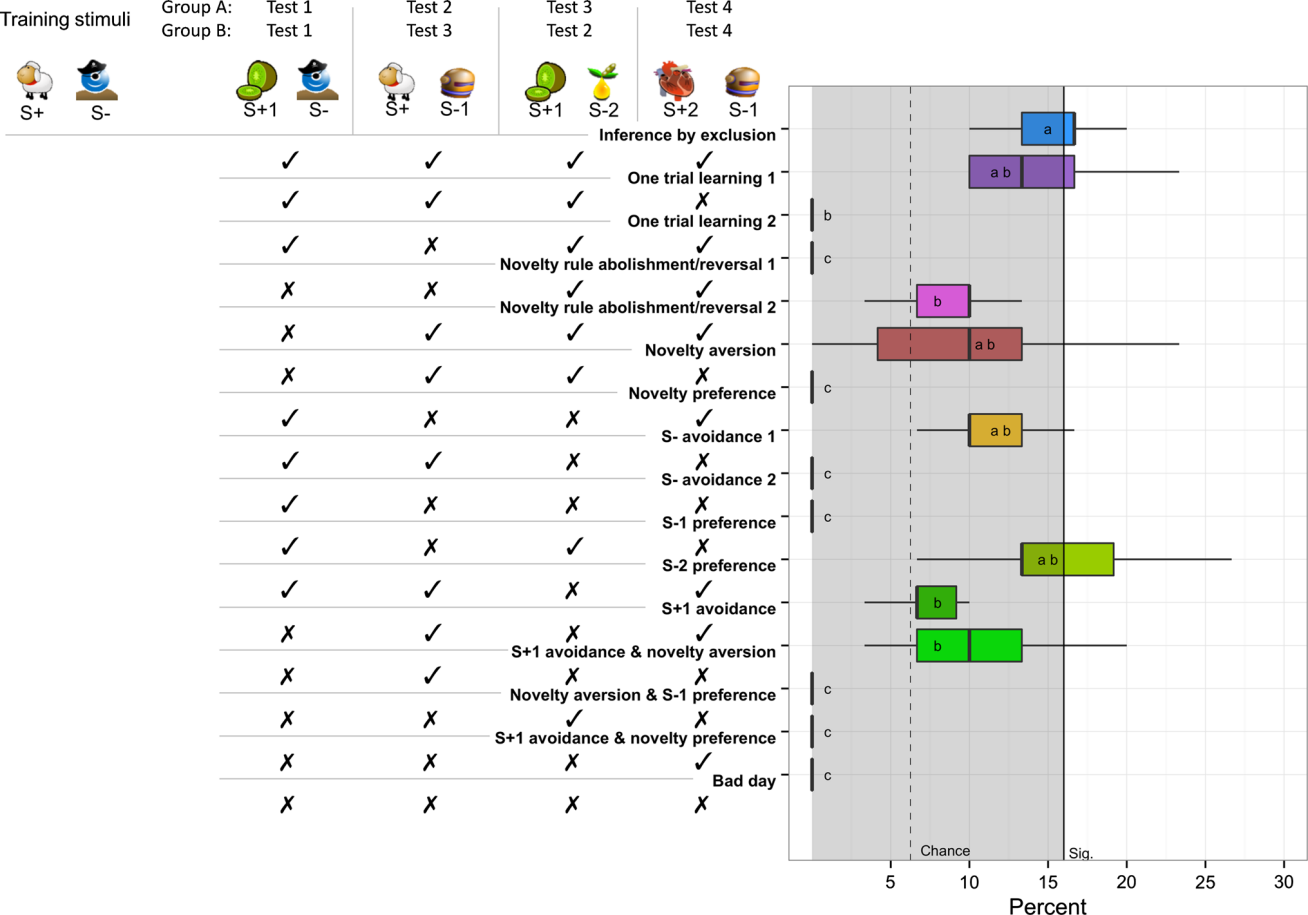
Schematic example of test trials and group-level results. Schematic representation of test trials with exemplary stimuli, as well as theoretical response predictions ordered with cognitively most demanding on top; + indicates rewarded stimuli; − indicates unrewarded stimuli; ✓ indicates correct; ✗ indicates incorrect choices, to the right percentage of response patterns employed at group level; *bold lines* indicate median values; *boxes* are spanning from the first to third quartiles; *whiskers* represent 95 % confidence intervals; outliers are not included; the *dotted line* and *grey area* indicate chance levels, while the *solid line* indicates significance as calculated by two-tailed binomial test

Test trials were presented consistently in a sequential order. Test trial one consisted of a novel positive stimulus (S + 1), while the S− remained the same as in the training trials. In test trial two, the S− was replaced with a novel unrewarded stimulus (S − 1) and presented with the S+ of the training trials, while the test trial three offered a choice of the novel rewarded stimulus of the test trial one (S + 1) and a completely novel unrewarded stimulus (S − 2). In test trial four, the novel unrewarded stimulus of test trial two (S − 1) was presented with a completely novel rewarded stimulus (S + 2). To investigate an effect of cognitive load, the two groups A and B differed in the sequence in which test trails were presented. Group A received test trials one to four in an ordinal order resulting in a test trial sequence: 1–2–3–4. Group B was presented with test trial three before test trial two, resulting in a test trial sequence of: 1–3–2–4 (see Fig. 1 for a schematic outline of a test session and sequence for each group). This resulted in corresponding test trials (test trials one and three, respectively, two and four) to follow in consecutive instances rather than intermixed, thus decreasing temporal space between them along with the possibility to be addressed sequentially rather than in parallel. The exact position for each test trial within a session was determined pseudo-randomly (trials 4 or 5 for the first test; trials 8 or 9 for the second test trial; trials 12 or 13 for the third test trial; trials 16 or 17 for the fourth test trial), to ensure that at least three training trials would be presented before the first test trial and before the end of a session, as well as to provide at least two training trials between each of the test trials.

### Data analysis

‘Response strategies’ were defined by attributing each possible response pattern the most parsimonious strategy (O’Hara et al. 2015a):

An instance of ‘inference by exclusion’ was only considered, if correct stimuli were chosen in all four test trials within a test session (see Fig. 1 for a graphic representation). These instances required individuals to either infer the contingency of the novel stimulus in the first test trial or simply avoid the S−. Further, in test trial three for group A (test trial two for group B) they had to avoid the novel stimulus (S − 2) and remain choosing the formerly rewarded S + 1. This is in principle the approach applied by Aust et al. (2008), controlling for correct responses in test trial one due to novelty preferences and simple avoidance of S−. However, individuals may have formed an association with the S + 1 as a result of being rewarded, which would qualify as one trial learning. Test trial two for group A (test trial three for group B) and test trial four are designed to control for such one trial learning. Here, the individuals should refrain from a novel unrewarded stimulus (S − 1) in test trial two for group A (test trial three for group B) and choose the known rewarded stimulus. This may be based on an avoidance of novelty, or simply a strong positive association with S+. However, in this case they have no direct information concerning the S − 1. Therefore, choosing the S + 2 in test trial four necessitates individuals to avoid S − 1 based on an inference, hence demonstrating the essence of inference by exclusion.

We assumed ‘one trial learning’ when individuals chose the unrewarded stimulus solely in the fourth test trial, as this indicates subjects fail to infer the contingency without direct feedback. We also considered it ‘one trial learning’ when the rewarded stimulus (S + 2) was chosen in the fourth test trial, after an initial response towards the novel stimulus (S − 1) in the second test trial.

A further possible strategy may have been guided by individuals transferring an acquired rule from the training that novel stimuli are always unrewarded. Therefore, they might avoid the novel S + 1 in the first test trial. However, if the subjects updated this rule and responded correctly in the following test trials or even reversed this rule, hence choosing the S − 1 instead of the original S+ in the second trial, we labelled this strategy ‘novelty rule abolishment/reversal’.

‘Novelty aversion’ was considered as choosing the stimulus which was more familiar to the kea, therefore responding correctly in the first and fourth test trials, but incorrectly in the second and third. ‘Novelty preferences’ were assumed if birds exhibited a reversed response pattern in test trials, by responding correctly in the second and third test trials, but incorrectly in the first and fourth test trials.

Multiple patterns that exhibited consistently avoiding rewarded stimuli or repeatedly choosing certain unrewarded stimuli—or combinations of such behaviours—were attributed to specific stimulus preferences or avoidances.

Finally, incorrect choices in all four test trials eluded any logical explanation on our part, which is why we inferred it must have been a ‘bad day’ for the individual.

We employed two-tailed binomial testing with a cumulative hypothesised probability (0.5 in test trial one × 0.5 in test trial two × 0.5 in test trial three × 0.5 in test trial four) of success of *p* = 0.0625. Generalised linear mixed effects models (GLMMs) with binomial error distribution and individual as a random factor were applied to investigate the effect of group, sex and session (in order to test whether certain strategies were acquired throughout the task) on the occurrence of response patterns exhibited above chance on a group level. As our sample size for young individuals is very low, we did not include age as a factor in the analysis. Statistical analyses were conducted in the R statistical package (R Development Core Team 2008), and for fitting models ‘lme4’ (Bates et al. 2014) was used. Tests were two-tailed, and alpha was set to 0.05.

## Results

**Fig. 2:**
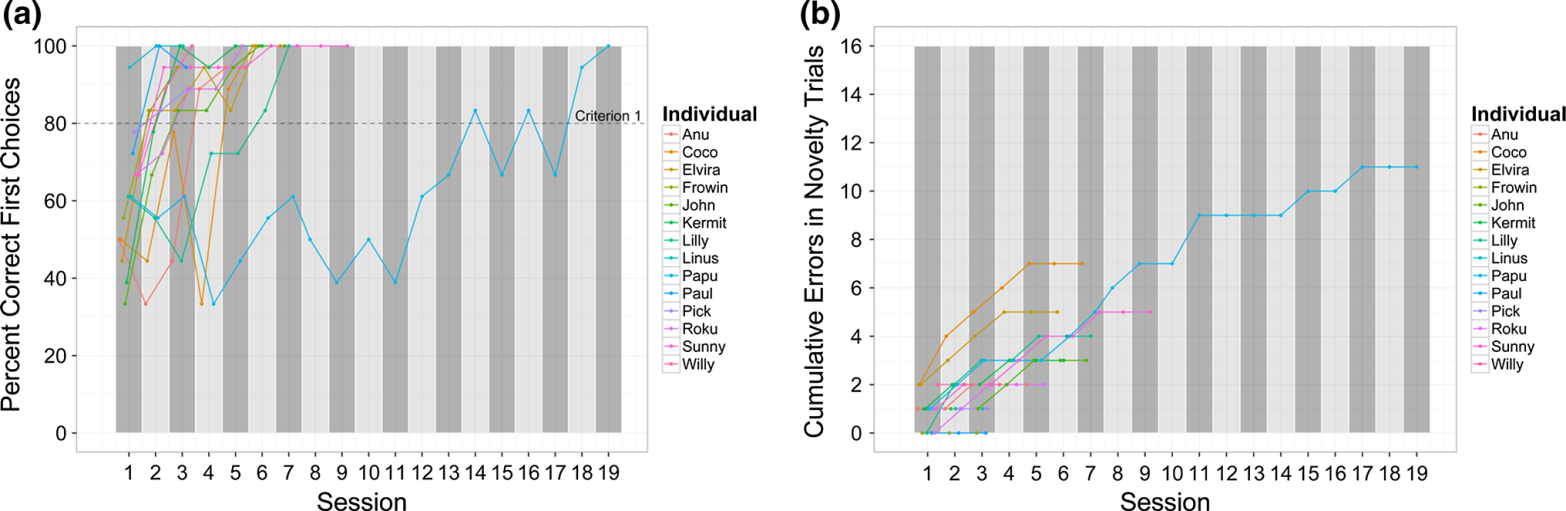
Performance in the training phase. **a** Learning curves for all individuals over the sessions, with the *dotted line* indicating the learning criterion of 80 % correct first choices (Criterion 1); longer the *lines* indicate more sessions required to reach criterion; **b** cumulative errors of novelty trials; a *steep inclination of lines* represents novelty responses in both novelty trials moderate inclination response to one novel stimulus per session and a *straight horizontal line* indicating no responses towards novel the stimuli (Criterion 2)

Seven individuals learned the discrimination of the baseline stimuli (Criterion 1: *M* = 3.7 sessions, ±0.43 SE) before ceasing to respond to the novel stimuli (Criterion 2: *M* = 6.1 sessions, ±0.70 SE, see Fig. 2). Five individuals reached both criteria simultaneously (*M* = 7.4 sessions, ±2.99 SE), and two kea quit responding to novel stimuli (*M* = 2 sessions, ±0.0 SE) before reaching the learning criteria (*M* = 3 sessions, ±0.0 SE), yielding a result bordering significance (Wilcoxon signed-rank test: *p* = 0.053, *r* = −0.365) for learning the discrimination faster (*M* = 4.9 sessions, ±1.14 SE) than refraining from exploring novel stimuli (*M* = 6 sessions, ±1.15 SE).

**Fig. 3:**
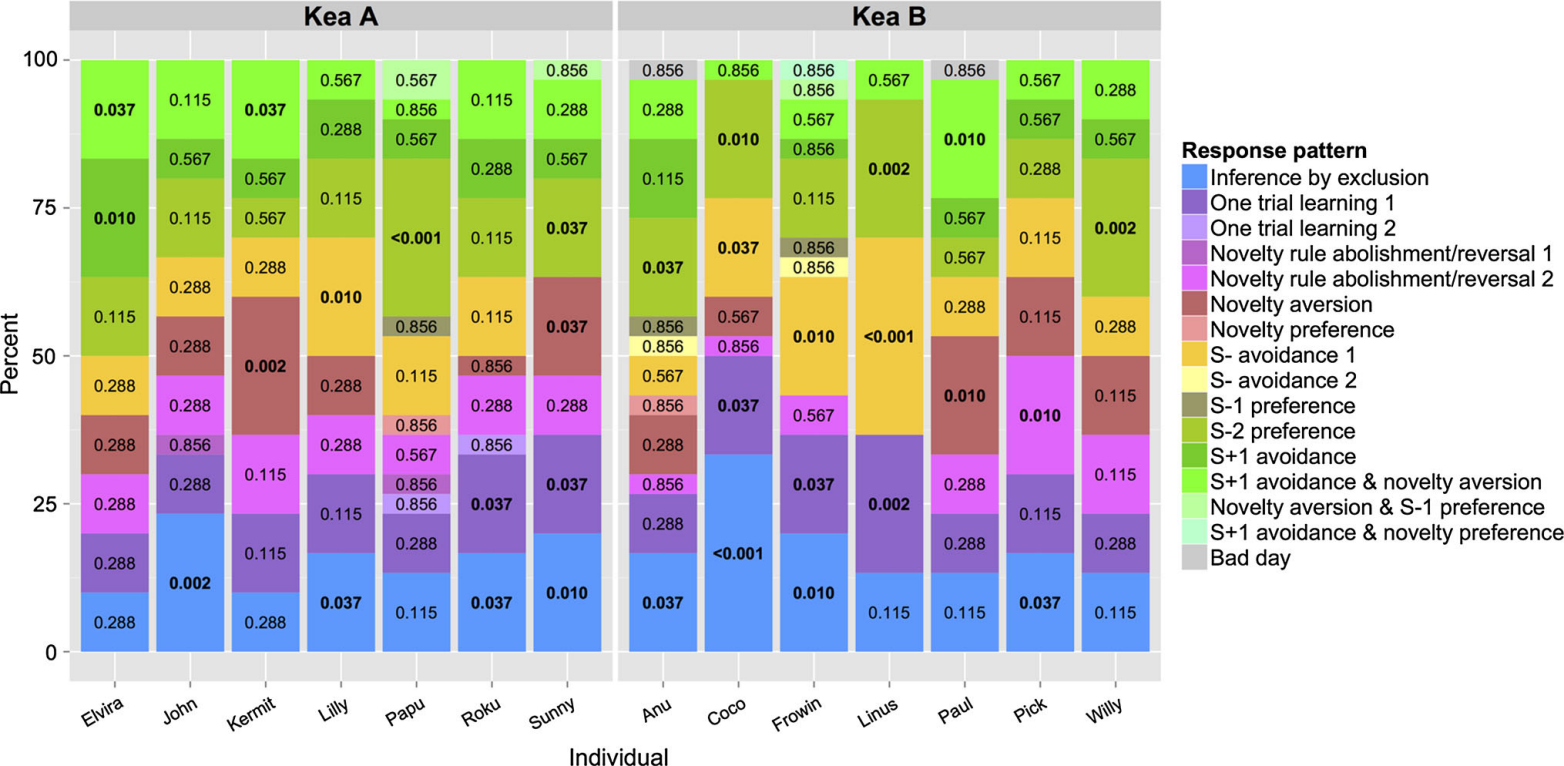
Distribution of strategies employed. Categorical strategies relied on by individuals of each group in the test; values enclosed in the *bar graphs* correspond to the adjusted probability of the amount of sessions with certain response patterns to occur by chance; significant values are printed in *bold*

On an individual level, we found eight individuals exhibiting responses suggestive of reasoning by exclusion significantly more often than expected by chance. Figure 3 displays the relative frequency with which each pattern was employed for each individual and values within the bars indicate the probability of this frequency occurring by chance. However, patterns of reasoning by exclusion did not occur as the sole strategy in seven of these eight individuals, as also up to three other strategies occurred above chance levels. Other strategies employed by most animals were one trial learning, avoiding the unrewarded baseline stimulus and stimulus preferences. Only one individual (John) seemed to rely solely on reasoning by exclusion. Individuals that did not exhibit reasoning by exclusion patterns above chance levels chose by avoiding novel stimuli, one trial learning and avoiding the S− in addition to stimulus preferences.

On a group level, the GLMM confirmed a significant intercept for occurrences of inferences based on exclusion (GLMM: *b* = −1.686, SE = 0.12, *Χ*^*2*^(1) = 73.89, *p* < 0.001), but excluded any effects of group (*Χ*^*2*^(1) = 0.55, *p* = 0.46), sex (*Χ*^*2*^(1) = 0.46, *p* = 0.497) or session (*Χ*^*2*^(29) = 31.08, *p* = 0.36). Similarly, one of the response patterns constituting one trial learning (GLMM: *b* = −1.925, SE = 0.13, *Χ*^*2*^(1) = 34.87, *p* < 0.001), one pattern suggesting S− avoidance (GLMM: *b* = −1.988, SE = 0.16, *Χ*^*2*^(1) = 19.79, *p* < 0.001) and preferences for S − 1 (GLMM: *b* = −1.783, SE = 0.12, *Χ*^*2*^(1) = 55.49, *p* < 0.001) occurred more often than predicted by chance on a group level (see Fig. 1). However, none of the above-mentioned factors had a significant effect on either of these strategies. When grouping only individuals that successfully responded according to inferring by exclusion and testing for frequencies of patterns, we found that patterns for ‘inference by exclusion’ (*M* = 6.33, ±0.57 SE) occurred significantly more often than patterns representing ‘one trial learning 1’ (*M* = 4.00, ±0.26 SE; Wilcoxon signed-rank test: *p* = 0.034, *r* = −0.53).

## Discussion

**Fig. 4:**
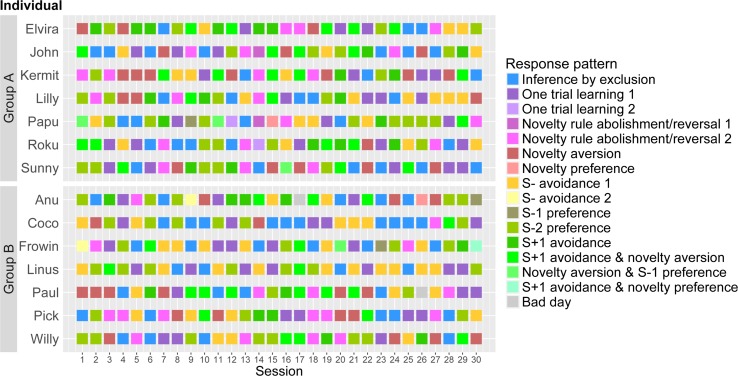
Individual distribution of response patterns over sessions. *Each square* represents colour-coded response patterns expressed by individuals of each group for each of the thirty test sessions; individuals are ordered within their respective experimental group

More than half of the subjects exhibited the ability to base their choices on inference by exclusion. They achieved this not only by considering direct feedback about a stimulus (from test trials one and three), but also by reasoning about the category of a stimulus based solely on the context in which it was presented and without any direct feedback (as in test trials two and four). This requires the individual to make an inference about the S − 1 in the absence of direct information about this stimulus. It is this lack of direct information that qualifies correct responses in the fourth task as exclusion based on an inference. One might argue that this pattern resulted from a combination of ‘one trial learning 1’ and random choice in the final test trial. However, considering that within individuals exhibiting significant exclusion performance, patterns for ‘inference by exclusion’ occurred significantly more often than the patterns for ‘one trial learning 1’, rendering this explanation unlikely. The fact that the number of sessions had no influence on the occurrence of this response pattern leads us to conclude that this strategy was not learned throughout the task (see Fig. 4). Thus, eight out of fourteen kea were able to spontaneously solve this abstract categorisation task by inferring the contingency of a novel stimulus through logical exclusion of the alternative.

It is interesting to note that the sequence in which the test trials were presented did not affect the performance of the individuals. Individuals of group A were required to store information about the S + 1 for longer and simultaneously pay attention to a novel stimulus with negative contingency (S − 1) in order to respond correctly in all test trials. Subjects of group B could apply the information about these stimuli in a more direct sequential manner, possibly implying a reduction in the cognitive load (Sweller 1988). Nevertheless, both groups performed equally well with respect to their exclusion skills. Therefore, it seems that an increase in cognitive load, in terms of working memory and parallel information storing, does not influence exclusion skills, at least not in this test design. An alternative explanation, as one reviewer pointed out, might be that the relatively subtle difference in procedure simply did sufficiently increase the cognitive load, and therefore, no effect was detectable. In this respect, future studies may address the effect of cognitive load more thoroughly by increasing the number of training trials between test trials to increase difficulty, or presenting corresponding test trials in direct succession, to decrease the load on the working memory.

However, the kea did not solely rely on inference skills in this task. On an individual level, we could show that novelty aversion, along with one trial learning, stimulus preferences and simply avoiding the S− also guided some individuals’ choices. Avoiding the S−, by forming a negative association with the unrewarded stimulus, may represent an efficient, low-cost strategy. Ecologically it seems adaptive to decrease responses towards a stimulus that does not provide any benefits (such as nutrition or information) or might even be harmful. Several studies on exclusion performance have discussed this alternative as a potential confound (e.g. Aust et al. 2008; Shaw et al. 2013; Nawroth et al. 2014), but conclusive controls remain rare (Call 2006; O’Hara et al. 2015a).

 Novelty aversion somewhat contradicts the kea’s natural predisposition to explore novel objects (Diamond and Bond 1999; Kubat 1992). One explanation for this result may be that three individuals (Kermit, Paul and Sunny) established a rule to avoid novel stimuli during the training phase, as these were never rewarded during the discrimination training. One might argue that this rule could contribute to the performance with respect to the inference by exclusion patterns; however, only one individual (Sunny) simultaneously showed significantly more occurrences of novelty avoidance and inference by exclusion than would be predicted by chance.

One trial learning can be considered a cognitively demanding strategy as it requires individuals to flexibly respond to novel stimuli based on one single feedback event. In particular, considering that responses to novel stimuli were (to some degree) extinguished during training highlights this flexibility applied in test trials.

Stimulus preferences and avoidances seem to occur in most tested subjects and pose a further low-cost strategy. Such preferences may arise simply through shared features with known stimuli, which are utilised by the individuals to attribute a positive or negative valence to a test stimulus. Overall, the large variance in strategies employed by different individuals in this task is indicative that cognitively less demanding mechanisms may override more ‘costly’ ones, whenever the opportunity is provided. What exactly the features are that provide these opportunities and when a certain threshold for such more simplistic classifications is passed remain subject to further research. Interestingly, however, none of the tested individuals seemed to have chosen purely randomly in the test trials, as would have been indicated by no preference for any particular response strategy.

As mentioned earlier, restriction in sample size of young subjects precluded an analysis of the effects of age on inference by exclusion. However, we would like to point out that both, juvenile and sub-adult, individuals (Papu and Paul) seem to have chosen based on perceptual cues, rather than employing inferences or one trial learning. In primates, it has been shown that exclusion abilities increase with age (Call 2006) suggesting that this ability may be subject to cognitive development. Although the small sample size of young individuals renders any conclusion from the current experiment highly speculative, this might open an interesting avenue for further studies, investigating if exclusion abilities in birds are developed with age as it appears to be the case in primates (Call 2006).

The holistic approach, allowing and considering multiple possible behavioural strategies underlying choice in the test trials, was essential to evaluate the effect of training to avoid novel stimuli in this study. Thus, we could show novelty aversion was not a strategy generally adopted, although some individuals did seem to have established this rule. Furthermore, the comparison of different strategies highlights the relative prevalence of inference by exclusion, as this appears to be the strategy that most often reached significance levels.

Taken together, the results indicate that kea are capable of inferring by exclusion, which contradicts earlier findings of Schloegl et al. (2009b). As has been hypothesised for other species (Mikolasch et al. 2012; Shaw et al. 2013), we assume that simpler cognitive mechanisms or predispositions (such as neophilia or stimulus enhancement) have masked previous attempts to reveal evidence for exclusion skills. In this respect, we would also expect the ravens, which showed reliable exclusion performance in the study by Schloegl et al. (2009b), to be guided by a range of different response strategies in this approach, similar to the kea. The fact that larger relative brain sizes are found in food-storing species has led some researchers to suggest that specialised feeding ecology is a driving force for brain evolution (Krebs 1990). However, by reversing the causality one might also assume that the evolution of large brains in relation to body size has allowed for cognitive abilities to emerge, which in turn provided some species with the cognitive framework to promote food-storing behaviour. There is growing evidence from psittacines and corvids, all with large relative brain sizes but differing in feeding ecology, to exhibit such inference by exclusion skills in different set-ups, such as African grey parrots (*Psittacus erithacus*, Mikolasch et al. 2011; Schloegl et al. 2012; Pepperberg et al. 2013), Clark’s nutcrackers (*Nucifraga columbiana*, Tornick and Gibson 2013), Goffin cockatoos (O’Hara et al. 2015a), New Caledonian crows (*Corvus moneduloides*, Jelbert et al. 2015) and ravens (Schloegl et al. 2009b). However, only inconclusive data are available for other members of these families, e.g. carrion crows (*Corvus corone corone*, Mikolasch et al. 2012), Eurasian jays (*Garrulus glandarius*, Shaw et al. 2013) and jackdaws (*Corvus monedula*, Schloegl 2011), and no inference by exclusion skills could be observed so far in the greater anis (*Crotophaga major*), itself not a member of either psittacines or corvids, in respect to the rejection of parasitic eggs (Riehl et al. 2015). This yields the consideration of reasoning by exclusion being a more fundamental cognitive trait, possibly coinciding with the evolution of large brains (Pepperberg et al. 2013). The hypothesis that it is a convergent adaption to feeding ecology (Schloegl et al. 2009a, b; Schloegl 2011; Mikolasch et al. 2012) has only recently been challenged by Shaw et al. (2013). Another compelling factor that might have contributed to a species’ capacity to infer by exclusion is the complexity of its social structure (Petit et al. 2015).

Here, we suggest a more holistic approach by considering multiple factors, such as social structure, migratory patterns, habitat complexity as well as specialised foraging strategies, as promoters of the evolution of advanced cognitive abilities (Tornick and Gibson 2013) in correlation with the evolution of larger brains (e.g. Harvey et al. 1980; Dunbar 1998; Reader and Laland 2002; Lefebvre et al. 2004; Connor 2007; Sol et al. 2010). In this sense, a mechanism would be adaptive that is capable of dealing with problems that require the same computation, but can be applied in different contexts. Hence, challenges in the physical and social domain may have selected for greater neuronal correlates that allowed establishing advanced cognitive capacities when confronted with similar problems in multiple domains.

The evidence of inference by exclusion in small-brained birds is far from compelling. There is some evidence for this ability in domestic chickens (*Gallus domesticus*, Hogue et al. 1996), graylag geese (*Anser anser*, Weiß et al. 2010), as well as pigeons (*Columba livia*, Wynne 1997). Pigeons could be ‘encouraged’ to select novel stimuli over defined ones, hence choosing by exclusion (Clement and Zentall 2003). However, this study could not show, given the applied set-up, whether pigeons would also be able to learn from this experience to infer unknown stimulus contingencies based on exclusion. The pigeon’s sensitivity to the testing protocol was demonstrated in a study by Aust et al. (2008) that applied the same (touch screen) procedure to several species comparatively. While some dogs and nearly all human participants showed evidence for learning by exclusion, all pigeon subjects failed.

The need for standardised testing protocols involving rigorous controls for alternative strategies has also been demonstrated in mammals. Dwarf goats (*Capra aegagrus hircus*) but not sheep (*Ovis orientalis aries*) performed above chance levels (Nawroth et al. (2014). However, the researchers could not exclude that individuals had acquired the simple strategy of avoiding the empty cup. The method of testing multiple concurrent strategies as presented here has already been successfully employed with Goffin cockatoos (*Cacatua goffini*) showing surprisingly similar response strategies (O’Hara et al. 2015a). We therefore suggest that the abstract categorisation task, a modified training procedure to discourage explorative behaviour before the test, and the specific sequence of tailored tests provide a suitable paradigm for inter-species comparisons of inference by exclusion abilities and competing response strategies. In general, we suggest shifting the focus from binary-outcome-driven tasks towards investigating category decisions and choice behaviour in a more holistic manner.
